# Obesity has no adverse effect on the survival of unicompartmental knee arthroplasty in a long‐term follow‐up of 16 years

**DOI:** 10.1002/jeo2.70426

**Published:** 2025-09-09

**Authors:** David Zhu, Regis Pailhe, Dany Mouarbes, Ali Alayane, Samy Saoudi, Hasnae Ben‐Roummane, Emilie Bérard, Etienne Cavaignac

**Affiliations:** ^1^ Chirurgie Orthopédique Traumatologique et Arthroscopique COTA, Hôpital Central Centre Hospitalier Régional Universitaire de Nancy (CHRU) Nancy France; ^2^ Centre Chirurgical Emile Gallé, Service de chirurgie orthopédique et traumatologique Centre Hospitalier Régional Universitaire de Nancy (CHRU) Nancy France; ^3^ Clinique Aguilera RAMSAY Santé Biarritz France; ^4^ Centre Hospitalier de Perpignan (CHP) Perpignan France; ^5^ Clinique Universitaire du Sport, Service de Chirurgie orthopédique et traumatologique Centre Hospitalier Universitaire de Toulouse (CHU) Toulouse France; ^6^ Research Methodological Support Unit (USMR), Department of Clinical Epidemiology and Public Health Toulouse University Hospital (CHU) Toulouse France; ^7^ Department of Clinical Epidemiology and Public Health, CERPOP, INSERM‐University of Toulouse III Toulouse University Hospital (CHU) Toulouse France

**Keywords:** arthroplasty, clinical outcome, obesity, survival, UKA

## Abstract

**Purpose:**

The role of obesity in unicompartmental knee arthroplasty (UKA) outcomes remains debated. The aim of this study is to clarify the impact of obesity on revision rates and functional outcomes of UKA in a long‐term follow‐up. The hypothesis was that body mass index and weight do not significantly affect the long‐term survival of UKAs.

**Methods:**

A retrospective study was conducted on 143 UKAs performed over a 16‐year period. Patients were stratified by BMI (<30 vs. ≥30 kg/m²) and weight (<82 vs. ≥82 kg). Kaplan–Meier survivorship analysis assessed the 16‐year survival rates across subgroups.

**Results:**

The 16‐year survival rates were comparable in the BMI subgroups (≥30 kg/m²: 78% (95% confidence interval [CI]: 60–88); <30 kg/m²: 84% [95% CI: 75–90]; *p* = 0.093) but demonstrated a significant difference in the weight subgroups (<82 kg: 85% [95% CI: 76–90]; ≥82 kg: 76% [95% CI: 59–87]; *p* = 0.045). Multivariate analysis, adjusted for confounders, showed no statistically significant impact of BMI (*p* = 0.202) or weight (*p* = 0.280) on the risk of revision. Functional outcomes, measured by knee society and self knee value scores, were unaffected by BMI at the final follow‐up.

**Conclusions:**

This study confirms that BMI and weight do not significantly influence the long‐term survival or functional outcomes of UKA, supporting its use in obese patients when appropriately indicated.

**Level of Evidence:**

Level IV, retrospective case series.

**Abbreviations**
BMI: body mass index
HKA: hip knee ankle angle
KSS: knee society score
OA: osteoarthritis
PCS: pain catastrophizing score
PE: polyethylene
SD: standard deviation
SKV: self knee value
SR: survival rate
TKA: total knee arthroplasty
UKA: unicompartmental knee arthroplasty
VAS: visual analogue score
WHO: World Health Organization

## INTRODUCTION

Unicompartmental knee arthroplasty (UKA) is now recognised as a popular and valid surgical treatment for end‐stage single‐compartment osteoarthritis (OA) [17]. While total knee arthroplasty (TKA) was traditionally the standard, UKA was introduced as a minimal procedure offering better long‐term outcomes when patients are properly selected [25]. Some studies have highlighted the advantages of UKA over TKA, including reduced intraoperative blood loss, lower transfusion requirements, shorter hospital stays and faster postoperative recovery of the knee function [31, 37]. These benefits were attributed to a less invasive alternative than TKA with preservation of a more natural knee kinematics and biomechanic [11]. However, survival rate of the implant tends to be higher than TKA [22]. At the end of the 20th century, Kozinn and Scott restricted UKA indications to patients weighing less than 82 kg and with a body mass index (BMI) below 30 kg/m² [15]. Over the past few decades, these limitations have been questioned. Numerous studies in literature have examined the mid‐to‐long‐term survival of UKA with follow‐up periods of up to 10 years based on BMI. However, their findings in obese patients remain inconsistent and contradictory. More recently, obesity, defined as a BMI ≥ 30 kg/m², has doubled globally since 1990, now affecting over 1 billion people, or 12% of the global population [28]. While the role of obesity in the development and progression of OA is well established, its impact on UKA outcomes, especially implant failure and revision rates remain controversial [7]. The aim of this study is to clarify the impact of obesity on revision rates and functional outcomes of UKA using the same patient cohort from the Cavaignac et al. study published in 2013, with an extended follow‐up period [4].

Our hypothesis is that BMI and weight do not affect the long‐term survival, and BMI does not affect the long‐term outcomes of UKA.

## PATIENTS AND METHODS

This retrospective cohort was conducted from 1990 to 2004 at a university hospital. All patients who underwent medial or lateral UKA procedure during this period were included. Patients lost to follow‐up at the last assessment were excluded due to insufficient data. In cases with bilateral UKAs, only the first procedure was included to avoid bias. Data were collected from the hospital's digital database and medical archive.

Implants used in all patients were the HLS Uni UKA (Tornier, Grenoble, France) and were all cemented with an all‐polyethylene (PE) tibial component. A medial subvastus approach was used in all cases. Operations were performed by three senior's surgeons at the Toulouse Rangueil University Hospital. An excessive BMI ≥ 30 kg/m^2^ was an absolute contraindication for one of the three surgeons according to Kozinn and Scott recommendations. Postoperative rehabilitation followed a standard protocol, including range of motion exercises, activation of the rectus femoris muscle, and gradual discontinuation of assistive walking devices.

Informed consent was obtained from all patients. Described research adhered to the tenets of the Declaration of Helsinki. The institutional review board considered this study as standard care and did not require further reviews.

### Outcomes

Implant survival was evaluated based on the need for UKA revision, defined as the addition, replacement, or removal of any prosthesis component. This included scenarios such as removal and replacement UKA in the same compartment, conversion to TKA using standard, revision or massive type of implants, or PE component replacement. Additionally, the implantation of a contralateral compartment UKA, osteotomy or osteosynthesis were also considered. The primary endpoint was to evaluate the survival rate of UKA in two subgroups according to BMI. Secondary endpoints included the survival rate stratified by weight and the influence of the BMI on functional outcomes.

Clinical and functional outcomes were assed at the final follow‐up from database at the last assessment using knee society score (KSS) and self knee value (SKV) [19, 27]. Patient were evaluated each year in standard care for knee function monitoring.

For deceased individuals, general practitioners and family members were approached to determine the date of death and to confirm whether any revision surgeries had been performed.

### Statistical analysis

Patient's characteristics were described using the following descriptive statistics for continuous variables: mean, standard deviation (SD), minimum and maximum value. For categorical variables, the frequency and percentage for each observed category are reported.

In order to identify potential confounding factors, Student's or Mann–Whitney's test, chi squared or Ficher's exact test were used to find significative association between baseline characteristics and BMI ≥ 30 kg/m^2^ or weight ≥82 kg.

Kaplan–Meier survival curves were utilised to evaluate overall survival based on BMI and weight. Survival curves for BMI and weight were compared using the log‐rank test. To control for potential confounding factors such as age or sex, multivariate analyses were conducted using a Cox proportional hazards model.

Functional scores were compared across BMI groups using either Student's or Mann–Whitney's test, depending on the normality of distributions and equality of variances between groups. Reoperation indications were detailed based on the number of cases and corresponding percentages, while mortality rate was reported along with the respective 95% confidence interval. The significance threshold for all analyses was set at *p* < 0.05. Statistical analyses were conducted using STATA Version 18.0 (StataCorp).

## RESULTS

**Figure 1:**
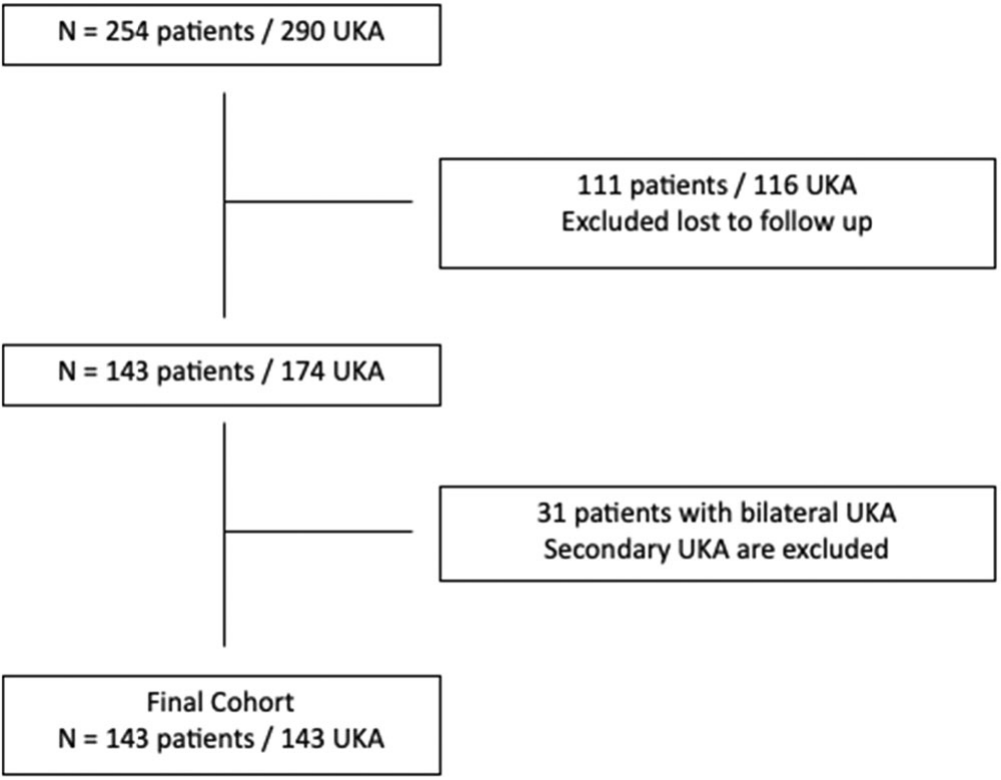
Flow chart diagram of the database screening. UKA, unicompartmental knee arthroplasty.

A total of 290 UKAs were performed in 254 patients, among these, 115 (111 patients) were lost to follow‐up, and 89 (70 patients) had died. The final cohort comprised 143 UKAs including 111 (77.6%) medial compartment and 32 (22.4%) lateral compartment in 143 patients (Figure 1).

The patients were divided into subgroups according to BMI (<30 or ≥30 kg/m^2^) and weight (<82 or ≥82 kg). The characteristics of the subgroups are showed on Table 1. In preoperative, for 29 knees (20.3%), there were degenerative changes in the contralateral tibiofemoral compartment (2 [1.4%] stage 1, 25 [17.5%] stage 2 and 2 [1.4%] stage 3 according to Ahlbäck and Iwano classification). Among the patients, only 4 (2.8%) had undergone prior anterior surgery in the same knee. Of these, 2 (1.4%) had tibial plateau osteosynthesis, while 1 (0.7%) underwent tibial varus osteotomy and another 1 (0.7%) underwent femoral valgus osteotomy.

**Table 1:** Characteristics of the subgroups according to BMI and weight.

Patients'characteristics	Total	Body mass index			Weight		
		<30 kg/m^2^	≥30 kg/m^2^	*p*‐value	<82 kg	≥82 kg	*p*‐value
Number	143	103	40		101	42	
Mean ± SD age, year	65.72 ± 10.09	66.83 ± 10.21	62.86 ± 9.30	**0.034** a	67.50 ± 9.90	61.44 ± 9.34	**<0.001** a
Gender ratio, men: women	1:2.57	1:2.12	1:4.7	0.82^b^	1:4.6	1:0.9	**<0.001** b
Compartment (%)				0.17^b^			0.29^b^
Medial	111 (77.6)	83 (74.8)	28 (25.2)		76 (68.5)	35 (31.5)	
Lateral	32 (22.4)	20 (62.5)	12 (26.8)		25 (78.1)	7 (21.9)	
Mean ± SD, body mass index, kg/m^2^	27.78 ± 4.38	25.60 ± 2.60	33.39 ± 2.75	**<0.001** a	26.14 ± 3.37	31.73 ± 4.02	**<0.001** a
Mean ± SD, weight, kg	74.92 ± 14.23	69.67 ± 11.63	88.82 ± 11.15	**<0.001** a	67.64 ± 8.72	92.40 ± 8.54	**<0.001** a
Unreplaced compartment osteoarthritis, *n* (%)	29 (20.3)	21 (20.4)	8 (20)	0.95^c^	24 (23.8)	5 (11.9)	0.108^c^
Femoropatellar osteoarthritis, *n* (%)	33 (23.1)	25 (24.3)	8 (20)	0.58^b^	26 (25.7)	7 (16.7)	0.24^b^
Mean ± SD, follow‐up, years	16.59 ± 7.58	16.71 ± 7.24	16.26 ± 7.47	0.74^a^	16.87 ± 7.34	15.90 ± 7.18	0.47^a^
Mean ± SD, preoperative HKA	176.17 ± 7.41	175.81 ± 7.25	177.12 ± 7.82	0.34^a^	176.32 ± 7.64	175.83 ± 6.91	0.72^a^
Anterior surgery, *n* (%)	4 (2.8)	3 (2.9)	1 (2.5)	0.73^c^	3 (2.9)	1 (2.5)	0.75^c^

Abbreviations: HKA, hip knee ankle; SD, standard deviation.

^a^ *p*‐value resulting from a student's *t*‐test.

^b^ *p*‐value resulting from a chi‐squared test.

^c^ *p*‐value resulting from a fischer exact test.

Regarding BMI, 103 (72%) patients had BMI < 30 kg/m^2^ while 40 (28%) had BMI ≥ 30 kg/m^2^. A significative age difference was observed with younger patients, more likely to have a BMI ≥ 30 kg/m^2^ (*p* = 0.034). Concerning weight, 101 (70.6%) patients weighed <82 kg and 42 (29.4%) weighed ≥82 kg. Statistical analysis revealed a significant association between a weight ≥82 kg and both male gender (*p* < 0.001) and younger age (*p* < 0.001).

The mean follow‐up period for the final cohort was 16.59 ± 7.58 years (7–31.33 years). The mean postoperative HKA was 178.4 ± 2.93° (170–194°). Out of 143 UKA, 29 (20.3%) patients underwent revision surgery. The mean postoperative time to revisions was 117.24 ± 94.71 months (1–300 months). In 24 (82.8%) cases, revision was involved by conversion to standard posterior stabilised TKA. Other revisions included: one revision TKA implants, one UKA in the other compartment, one switch of the PE, one valgisation osteotomy and one osteosynthesis were used (3.4% each). Regarding the indications for revision, 14 knees (48.3%) had contralateral OA decompensation, five cases (17.2%) involved unexplained pain, four cases (13.8%) were due to aseptic loosening and two (6.9%) were associated with periprosthetic fracture. Additionally, there was one case (3.4%) patient for each of these indications: femoral component impingement, tibial component impingement, polyethylene wear and excessive varus malalignment. The mean time of revision is summarised in Table 2.

**Table 2:** Indication of the revisions with reoperative mean time.

Indication of revision	*N* (%)	Mean time ± SD (year)
Contralateral decompensation	14 (48.3)	9.56 ± 6.15
Unexplained pain	5 (17.2)	10.47 ± 9.65
Aseptic loosening	4 (13.8)	9.43 ± 9.81
Periprosthetic fracture	2 (6.9)	8.58 ± 11.99
Femoral component conflict	1 (3.4)	4.07 ± NA
Tibial component conflict	1 (3.4)	2.89 ± NA
Wear of the PE	1 (3.4)	4.62 ± NA
Excessive varus	1 (3.4)	23.09 ± NA

Abbreviations: NA, Non applicable; PE, polyethylene; SD, standard deviation.

Regarding to the mortality rate at the end of follow‐up in the cohort, 49% (95% CI, 40.5–57.4) (70 UKA) patients died, of which three patients (2.1%) undergoing revision surgery prior to death.

**Figure 2:**
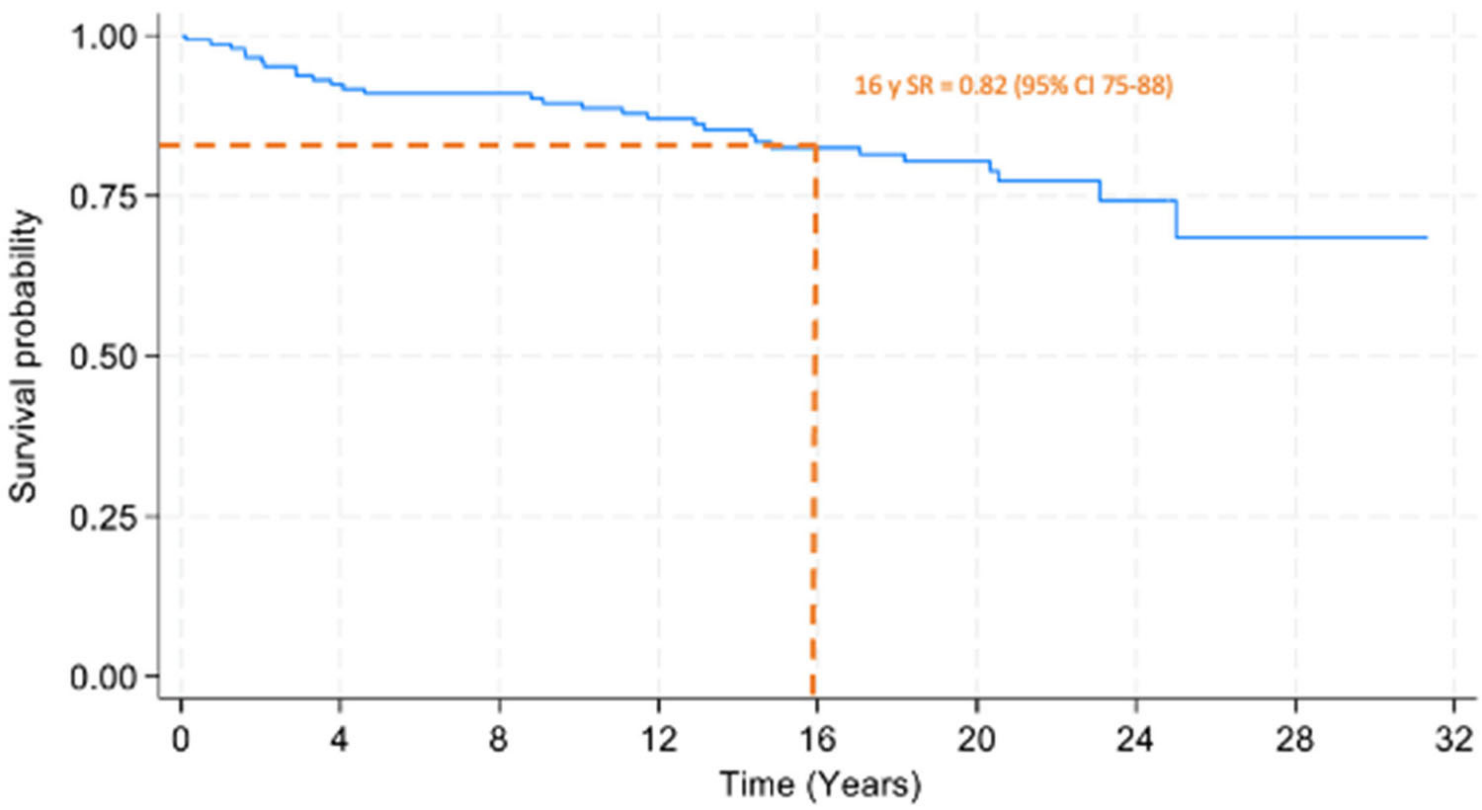
Kaplan–Meier survivorship curve for the entire cohort (16 years SR, 16‐year survival rate with 95% confidence interval).

**Figure 3:**
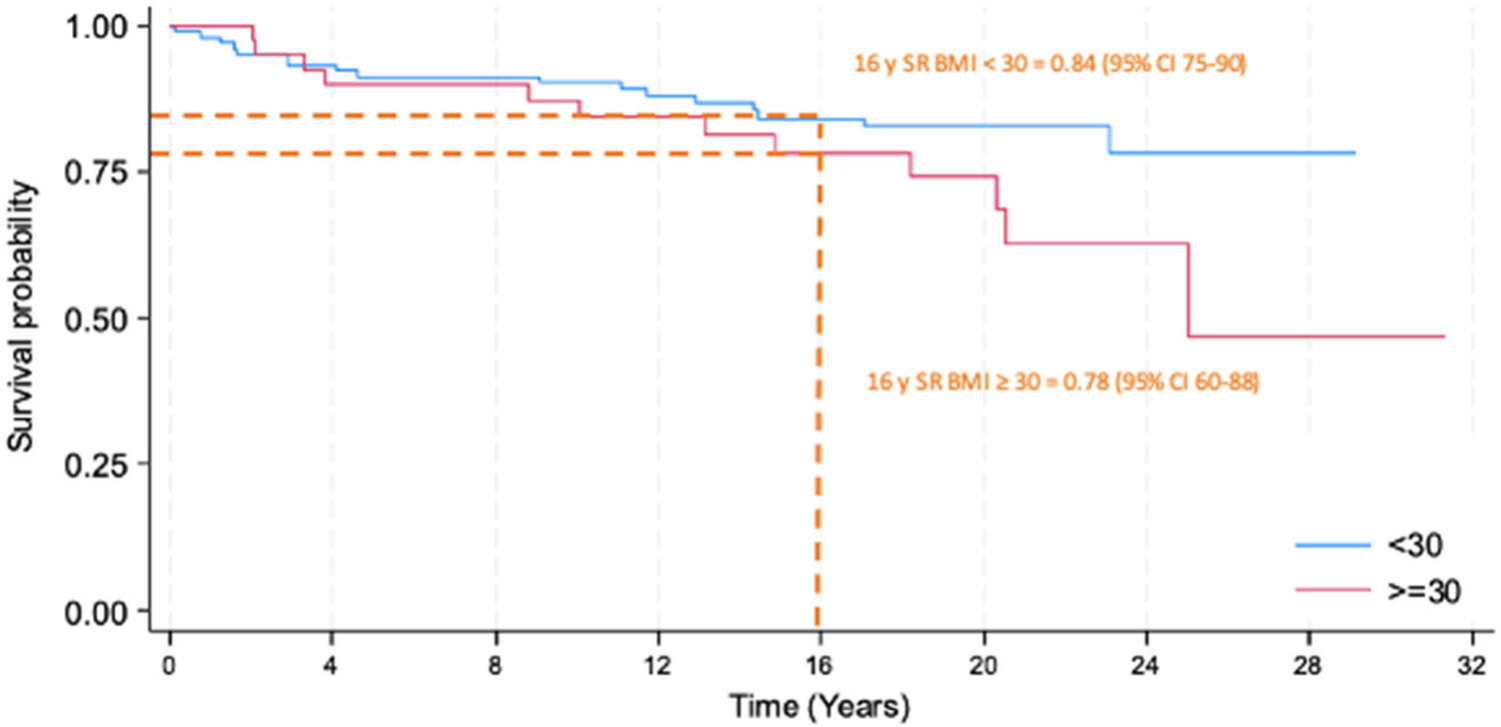
Kaplan–Meier survivorship curve of the subgroups based on body mass index (BMI). Blue line represents the curve for BMI < 30 kg/m^2^, the red line represents the curve for BMI ≥ 30 kg/m^2^, the dotted line represents the 16‐year survival rate (16 years SR, 16‐year survival rate with 95% confidence interval).

**Figure 4:**
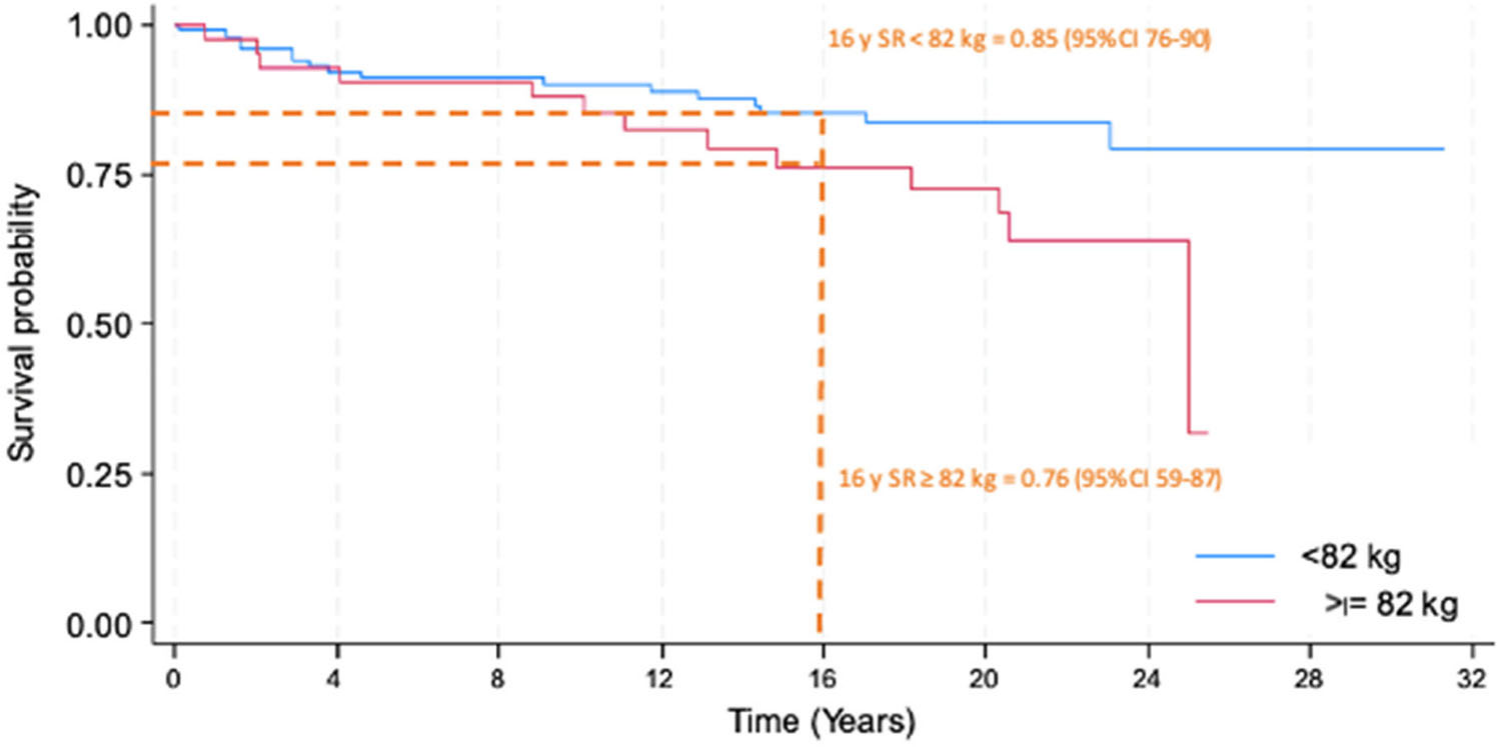
Kaplan–Meier survivorship curve of the subgroups based on weight. Blue line represents the curve for <82 kg, the red line represents the curve for ≥82 km, the dotted line represents the 16‐year survival rate (16 years SR, 16‐year survival rate with 95% confidence interval).

The 16‐year survival rate (SR) for the entire cohort was 82% (95% CI: 75–88), with no significant difference between the BMI subgroups <30 kg/m² and ≥30 kg/m² (*p* = 0.093), showing SR of 84% (95% CI: 75–90) and 78% (95% CI: 60–88), respectively (Figures 2 and 3). In contrast, when stratified by weight (<82 and ≥ 82 kg), the SR showed a statistically significant difference in univariate analysis (*p* = 0.045), with SR value of 85% (95% CI: 76–90) and 76% (95% CI: 59–87), respectively (Figure 4).

Due to preoperative statistical differences in age between the BMI subgroups, and in age and sex between the two weight subgroups, an adjusted multivariate analysis was performed. After adjustments, no significant differences were observed in either group based on BMI (*p* = 0.202) or weight (*p* = 0.280). The statistical analysis is showed on Table 3.

**Table 3:** Multivariate analysis adjusted for age for the two BMI subgroups, and for age and sex for the two weight subgroups.

	*N*	Revision surgery	Hazard ratio	95% CI	*p*‐value
BMI					
<30	103	17	1		
≥30	40	12	1.63	[0.77, 3.43]	0.202
Age					
<70 years	85	24	1		
≥70 years	58	5	0.38	[0.14, 1.02]	0.056
Weight					
<82 kg	101	16	1		
≥82 kg	42	13	1.57	[0.69, 3.55]	0.280
Age					
<70 years	85	24	1		
≥70 years	58	5	0.43	[0.16, 1.17]	0.098
Sex					
Male	40	12	1		
Female	103	17	0.73	[0.32, 1.68]	0.462

Abbreviations: 95% CI, 95% confidence Interval; BMI, body mass index.

At the end of follow‐up, 47 patients (32.9%) were alive without requiring revision. The mean follow‐up for this population was 22.9 + 2.78 years (19.5–31.3 years). The mean functional scores at the last assessment were 85.9 ± 13.7 (50–100) for the KSS knee score, 67.9 ± 22.8 (20–100) for the KSS function score, and 72.4 ± 16.6 (10–100) for the SKV score. No significant differences in functional scores were observed between the two BMI subgroups (<30 and ≥30 kg/m^2^). The detailed relationships are presented in Table 4.

**Table 4:** The relationships between BMI and functional scores at final follow‐up.

	BMI			
	<30	≥ 30	Total	*p*‐value
	(*N* = 40)	(*N* = 7)	(*N* = 47)	
Score KSS knee				
Mean (min–max)	85.12 (50–100)	90.71 (77–95)	85.96 (50–100)	0.720^b^
Score KSS function				
Mean (min–max)	66.00 (20–100)	78.57 (50–90)	67.87 (20–100)	0.232^b^
Score SKV				
Mean (SD)	71.88 (10–100)	75.71 (50–90)	72.45 (10–100)	0.578^a^

Abbreviations: BMI, body mass index; KSS, knee society score; max, maximum; min, minimum.

^a^ *p*‐value resulting from a student's *t*‐test.

^b^ *p*‐value resulting from a Mann–Whitney's test.

## DISCUSSION

The main finding of this study corroborates previous research, which involved the same cohort. It confirms that an increased BMI does not negatively impact the survival rates of UKA, particularly with the extended follow‐up period in this study. The analysis revealed no statistically significant differences in the 16‐year survival rates or the 22.8‐year clinical outcomes between the two BMI subgroups, underscoring the viability of UKA regardless of BMI.

UKA has gained popularity due to its strong long‐term survivorship and functional outcomes [5]. However, the role of patient weight or BMI in the success of UKA remains a contentious issue in literature. High BMI (≥30 kg/m²) has traditionally been seen as a contraindication due to concerns about wear and prosthesis survival [15]. Advancements in techniques and prosthesis designs suggest this view may be overly restrictive, limiting UKA's adoption. As a result, surgeons are reconsidering the impact of obesity on UKA outcomes, with studies showing conflicting results. While many have identified obesity as a negative factor influencing UKA outcomes [13, 14, 24, 36, 38], others have provided evidence supporting its viability in this patient population [1, 4, 9, 10, 20, 21, 29, 30].

Several studies have raised concerns about UKA in obese patients citing higher implant failure and reduced survivorship. Nettrour et al. reported a fivefold increase in revision rates for morbidly obese patients (BMI ≥ 40) at 3.5 years after examining 152 UKAs [24]. Vaz et al. included 468 UKA patients with an average follow‐up of 8.5 years, demonstrating that obesity and ACL deficiency significantly raised the risk of revision [36]. Kandil et al. used national databases to analyse 15,770 UKA and found that obese patients had nearly double the revision rate compared to nonobese patients over an average of 7 years [14]. Jeschke et al. analysed nationwide data from Germany, including 20,946 UKAs, and reported that obesity, depression and complicated diabetes significantly negatively impacted 5‐year UKA survival rates [13]. Xu et al. prospectively followed 184 UKA patients for 10 years and found that obesity was a predictor of poorer clinical outcomes and increased revision rates [38].

In our study, weight was a secondary point in assessing the risk of UKA failure. Univariate analysis revealed significantly higher revision rate in the >82 kg group (*p* = 0.045). Preoperative demographic data and analysis showed significantly younger patient in the heavier group (*p* < 0.001). This trend is also observed in national USA epidemiological data, which indicate that obesity prevalence increased before and decreased after 60 years old [32]. Moreover, several studies have found a younger age <50 years old witch are more physically active is associated with significantly higher rate of UKA aseptic loosening revision [6, 33]. However, multivariate analysis after adjusting for these demographic variables found no further any statistically significant difference in survival rates between the two weight groups.

On the other hand, several studies challenge the belief that high BMI negatively impacts UKA outcomes, reporting no reduction in survival rates for obese patients. Molloy et al. found no survival differences in 1000 Oxford UKAs over 10 years [21]. Purcell et al. reported no significant revision rate differences across BMI categories in 1167 UKAs at 7.2‐year follow‐up [30]. Giordano et al. observed a 97% 5‐year survival rate in 101 lateral UKAs, with no BMI‐related differences [9]. Affatato et al. found obese and nonobese patients had comparable outcomes in a multicenter study of 4904 UKAs at 6.5‐year follow‐up [1]. Other studies similarly found no significant BMI‐related differences in revision or outcomes, including Mohammad et al., Plate et al. and Gregor et al. [10, 20, 29].

Our finding revealed no statistical difference in multivariate analysis based on BMI and weight with 30 mg/kg^2^ and 82 kg as thresholds at 16 years survival corroborate with these studies. Furthermore, the 82% survival rate at 16 years of the entire cohort appears satisfactory when compared with the existing literature. To our knowledge, this study is the longest follow‐up in the literature.

As for meta‐analyses examining the impact of BMI on UKA, varying conclusions have been published. Mushabi et al. reviewed nine studies with 4621 UKAs and found no significant outcome differences with higher BMI, despite a trend towards higher revision rates [23]. Agarwal et al. analysed 30 studies with 80,798 UKA patients and reported no significant differences in complications or revision rates between obese and nonobese patients [2]. Similarly, Van der List et al. found a slight, nonsignificant increase in revisions in obese patients across 21,204 cases after analysing six cohort studies and two registries [34]. Lua et al. observed a trend toward higher revisions in obese patients across 42,434 UKAs from 25 studies, but these differences were not statistically significant [18]. However, other studies highlighted potential risks. Campi et al. reported a higher all‐cause revision rate for patients with a BMI > 30 in their meta‐analysis of 11 studies involving 40,753 patients [3]. Jia Hui et al. analysed 12 studies with 21,173 patients and found that BMI > 30 had a higher risk ratio for all‐cause revisions compared to nonobese patients [26]. Vasso et al. reviewed 17 studies including 43,845 patients and identified that both obesity and severe obesity (BMI > 35) were associated with significantly higher revision rates and lower implant survival rates [35].

Most studies on UKA in obese patients are limited by small sample sizes, few revisions, short follow‐up, or varied prostheses, with most having a mean follow‐up of 10 years or less. However, even 10 years may not suffice to assess implant survivorship. To our knowledge, only one long‐term study with a follow‐up exceeding 10 years examined the impact of BMI on fixed‐bearing UKA outcomes as our study. It reported a statistically significant difference (*p* = 0.017) in survivorship, with a 74.2% survival rate in obese patients (BMI ≥ 30 kg/m²) versus 92.4% in controls after 15 years, based on 70 UKAs [16].

In our study, the World Health Organization (WHO) guidelines was followed, using a BMI of 30 kg/m² to differentiate normal weight and obese groups [12]. A significant age difference at the time of UKA was found, with obese patients typically developing OA at a younger age, as reported in previous research [8]. Our cohort had a mean follow‐up of 16.5 years, representing the longest follow‐up reported in the literature, with a maximum of 31.3 years. After adjusting for age, no significant differences in survivorship were found between BMI groups (*p* = 0.202). Additionally, using Kozinn and Scott's 82 kg weight threshold [15], no statistical difference in revision rates was observed between the weight‐based subgroups after adjusting for age and sex. Only two subgroups of BMI and weight were set on this study due to insufficient patients with very excessive BMI and weight. Our findings may not be generalised to extreme BMI and weight because of this lack of data. Moreover, previous studies focusing on morbidly obese patients (BMI > 40 kg/m²) have reported conflicting results regarding revision rates [1, 24, 35].

Regarding the reasons for revision, ‘ongoing pain’ was the most common cause of revision in Gregor et al.'s study notably higher in obese patients [10]. Other studies identified OA progression in the contralateral compartment as the leading cause of failure [24, 30]. In our study, within the BMI > 30 kg/m² subgroup, revisions included eight for contralateral OA, two for unexplained pain and two for aseptic loosening. Contralateral OA progression was the most common cause of revision overall, accounting for 14 patients out of 29 (48%), particularly in the obese subgroup, accounting for eight patients out of 12 (67%). Failure may reflect natural progression of OA or changes in the biomechanical forces acting on the unreplaced compartment after UKA. In 2013, Cavaignac et al. identified malalignment as a revision risk factor in both obese and nonobese patients [4].

Functional scores in studies vary due to follow‐up durations and BMI classifications. Xu et al. found lower KSS, oxford knee score (OKS) and pain catastrophizing score (PCS) in obese patients at 10 years [38], while Molloy et al. reported significant OKS improvements with increased BMI [21]. Lee et al. found similar outcomes between BMI groups [16]. In Vasso et al.'s meta‐analysis, no significant effect of obesity on functional outcomes was noted, though KSS was lower in severely obese patients [35]. Lua et al.'s meta‐analysis found greater OKS improvement in obese patients but no differences in KSS or visual analogue score (VAS) scores [18]. In our cohort, after an average 22.9‐year follow‐up, the longest reported in literature, no significant differences in functional outcomes were found between obese and nonobese patients, with KSS and SKV scores unaffected by BMI in 47 patients without revision.

This study has limitations due to its retrospective design, single‐centre data collection and involvement of three surgeons, which may affect generalisability. Furthermore, despite the monitoring of UKA in our department, the substantial number of lost to follow‐up in this study may introduce an attrition bias, leading to an underestimation in the revision rate or functional outcomes. This may reduce the statistical power analysis and limit the extrapolation of the findings. Moreover, it combined medial and lateral UKAs, with most being medial, and analysed only two BMI subgroups due to insufficient patients with BMI ≥ 35 kg/m². This insufficient data may also limit the interpretation of the findings on extreme obesity. Despite these limitations, this study is noteworthy for its large cohort size and the extensive long‐term follow‐up period, providing important insights into the influence of BMI on UKA survival. In addition to implant survival, functional improvements even in obese patients persist over the time, with good results in our cohort at over 30 years.

## CONCLUSION

Advancements in UKA designs have changed historical views on weight and obesity as contraindications. Our findings show that less obese patients have similar long‐term survival rates and functional outcomes as nonobese patients, suggesting that obesity should not be a contraindication for UKA when indicated. This obstacle should be today, reconsidered and the old thresholds reevaluated with further large cohort studies.

## AUTHOR CONTRIBUTIONS

**David Zhu**: Conceptualisation; data curation; writing original—review and editing. **Regis Pailhe**: Review and editing. **Dany Mouarbes**: Review and editing. **Ali Alayane**: Review and editing, **Samy Saoudi**: Data Curation, **Hasnae Ben‐Roummane**: Formal analysis. **Emilie Bérard**: Formal analysis; review and editing. **Etienne Cavaignac**: Conceptualisation; review and editing; validation.

## CONFLICT OF INTEREST STATEMENT

All authors certify that they have no affiliations with or involvement in any organisation or entity with any financial interest or nonfinancial interest in the subject matter or materials discussed in this manuscript.

## ETHICS STATEMENT

The authors have nothing to report.

## Data Availability

The data that support the findings of this study are available from the corresponding author upon reasonable request.
